# Side-by-side comparison of the two widely studied GRPR radiotracers, radiolabeled NeoB and RM2, in a preclinical setting

**DOI:** 10.1007/s00259-023-06364-4

**Published:** 2023-08-16

**Authors:** T. S. T. Damiana, P. Paraïso, C. de Ridder, D. Stuurman, Y. Seimbille, S. U. Dalm

**Affiliations:** 1 Department of Radiology & Nuclear Medicine, Erasmus Medical Center, Rotterdam, The Netherlands; 2 Department of Experimental Urology, Erasmus Medical Center, Rotterdam, The Netherlands

**Keywords:** GRPR, NeoB, RM2, Peptide receptor radionuclide therapy, Prostate cancer

## Abstract

**Introduction:**

NeoB and RM2 are the most investigated gastrin-releasing peptide receptor (GRPR)–targeting radiotracers in preclinical and clinical studies. Therefore, an extensive side-by-side comparison of the two radiotracers is valuable to demonstrate whether one has advantages over the other. Accordingly, this study aims to compare the in vitro and in vivo characteristics of radiolabeled NeoB and RM2 to guide future clinical studies.

**Method:**

The stability of the radiolabeled GRPR analogs was determined in phosphate buffered saline (PBS), and commercially available mouse and human serum. Target affinity was determined by incubating human prostate cancer PC-3 cells with [^177^Lu]Lu-NeoB or [^177^Lu]Lu-RM2, + / − increasing concentrations of unlabeled NeoB, RM2, or Tyr^4^-bombesin (BBN). To determine uptake and specificity cells were incubated with [^177^Lu]Lu-NeoB or [^177^Lu]Lu-RM2 + / − Tyr^4^-BBN. Moreover, in vivo studies were performed to determine biodistribution and pharmacokinetics. Finally, radiotracer binding to various GRPR-expressing human cancer tissues was investigated.

**Results:**

Both radiotracers demonstrated high stability in PBS and human serum, but stability in mouse serum decreased substantially over time. Moreover, both radiotracers demonstrated high GRPR affinity and specificity, but a higher uptake of [^177^Lu]Lu-NeoB was observed in in vitro studies. In vivo, no difference in tumor uptake was seen. The most prominent difference in uptake in physiological organs was observed in the GRPR-expressing pancreas; [^177^Lu]Lu-RM2 had less pancreatic uptake and a shorter pancreatic half-life than [^177^Lu]Lu-NeoB. Furthermore, [^177^Lu]Lu-RM2 presented with a lower tumor-to-kidney ratio, while the tumor-to-blood ratio was lower for [^177^Lu]Lu-NeoB. The autoradiography studies revealed higher binding of radiolabeled NeoB to all human tumor tissues.

**Conclusion:**

Based on these findings, we conclude that the in vivo tumor-targeting capability of radiolabeled NeoB and RM2 is similar. Additional studies are needed to determine whether the differences observed in physiological organ uptakes, i.e., the pancreas, kidneys, and blood, result in relevant differences in organ absorbed doses when the radiotracers are applied for therapeutic purposes.

**Supplementary Information:**

The online version contains supplementary material available at 10.1007/s00259-023-06364-4.

## Introduction

**Fig. 1:**
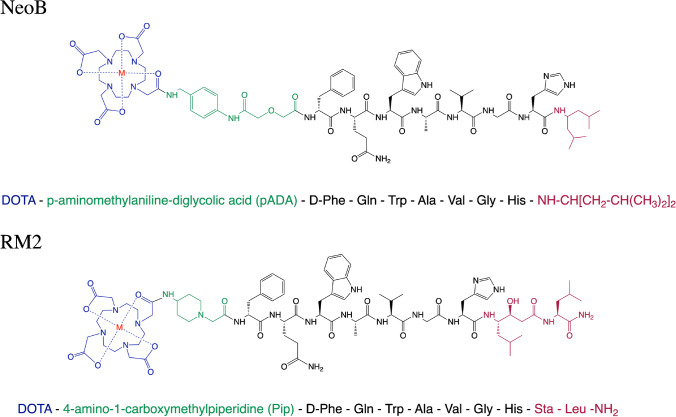
Chemical structures of NeoB and RM2. Blue: chelator, green: linker, black (and red) targeting moiety (and c-terminus)

The gastrin-releasing peptide receptor (GRPR) is a G protein–coupled receptor belonging to the family of bombesin (BBN) receptors. The receptor is expressed in the pancreas, and very low levels have also been reported in the colon, prostate, and some regions of the central nervous system. Stimulation of GRPR can induce several pharmacological and biological responses, such as smooth muscle contraction and hormone secretion [1]. Next to the physiological expression, overexpression of GRPR has been reported in various cancer types, including lung-, breast- (BC), pancreatic-, prostate- (PCa), head and neck cancer, and neuroblastomas/glioblastomas [2–4]. Due to its overexpression in these cancer types, radiotracers targeting the GRPR have been developed for peptide receptor radionuclide imaging and therapy (PRRT) [5–7]. Both GRPR-targeted analogs with agonistic and antagonistic properties have been developed and evaluated. GRPR antagonists have shown superior tumor uptake and pharmacokinetic properties compared to agonists. Furthermore, the use of GRPR antagonists prevents adverse side effects, e.g., nausea and diarrhea, caused by receptor activation that was previously observed with agonists [8–10]. Radiolabeled GRPR antagonists studied during the past years include, e.g., RM1, RM2, SB3, NeoB, ProBOMB, and RM26, of which NeoB and RM2 are the two most investigated GRPR-targeting radiotracers in preclinical and clinical studies [11–16]. The structural difference between NeoB and RM2 can be found at the C-terminal sequence and the linker that connects the chelator to the targeting moiety (Fig. 1). The C-terminus of NeoB consists of (His^12^)-NH-CH[CH_2_-CH(CH_3_)_2_], and the universal DOTA-chelator is connected to a *p*-aminomethylaniline-diglycolic acid (*p*ADA) linker at the N-terminus [14, 17, 18]. RM2 has a (His^12^)-Sta^13^-Leu^14−^NH_2_ sequence at the C-terminus, and the N-terminal DOTA-chelator is conjugated to a 4-amino-1-carboxymethylpiperidine (Pip) linker [19].

Radiolabeled NeoB and RM2 have demonstrated promising results in preclinical and clinical studies [17, 18, 20, 21]. Currently, a phase I/IIa clinical trial (NeoRay, NCT03872778) is ongoing in which the safety, tolerability, pharmacokinetics, distribution, radiation dosimetry, and anti-tumor activity of [^177^Lu]Lu-NeoB are investigated in patients with tumors known to overexpress GRPR. Another clinical trial is planning to investigate different doses of [^177^Lu]Lu-NeoB in combination with radiotherapy and temozolomide in newly diagnosed patients with glioblastoma (NCT05739942). Regarding RM2, two ongoing trials, i.e. NCT03113617 and NCT02624518, using gallium-68 labeled RM2 PET/CT and PET/MRI, respectively, are evaluating its potential to detect PCa.

An extensive side-by-side comparison of the radiotracers can be valuable to demonstrate whether one has specific advantages over the other to guide future (clinical) studies. Therefore, the current study aims to accurately compare stability, target affinity, specificity, biodistribution, and pharmacokinetics of radiolabeled NeoB and RM2 to guide future clinical studies.

## Material and methods

### Chemistry and radiolabeling

NeoB and RM2 were synthesized as described in Supplementary material 1. Both peptides were labeled with lutetium-177 (LuMark®, IDB Holland, The Netherlands) for all in vitro cell studies and in vivo studies. For autoradiography studies, the peptides were labeled with indium-111 (Covidien, The Netherlands) [22]. Lutetium-177 or indium-111 was added to NeoB and RM2, together with water (109 µL), sodium acetate (1 µL, 2.5 M, pH 4.5), ascorbic/gentisic acids (10 µL, 50 mM) and l-methionine (10 µL, 50 mM) to prevent radiolysis [23]. The mixtures were incubated at 90 °C for 20 min and then left to cool down for 5 min. For all in vitro assays and autoradiography studies, the peptides were labeled with a molar activity of 20 MBq/nmol. The in vivo studies were performed with a molar activity of 60 MBq/nmol. The radiolabeling yield (RCY) was determined by instant thin-layer chromatography on silica-gel-impregnated glass fiber sheets (Agilent, The Netherlands) eluted with a solution of sodium citrate (0.1 M, pH 5)), in order to monitor the completion of the labeling reactions. Unbound lutetium-177 was complexed to diethylenetriaminepentaacetic acid (DTPA), which is known to be excreted immediately after in vivo injection in mice [24]. Furthermore, data was corrected for RCY by multiplying counts per minute with the determined correction factor (100%/RCY%). Additionally, Kolliphor HS 15 (2 mg/ml; Sigma Aldrich, USA) was added to all radiolabelings to prevent sticking of the peptides. The radiochemical purity (RCP) was determined by radio-high pressure liquid chromatography (HPLC) (LC/MS 1260 Infinity II system, Agilent, The Netherlands) (Supplementary material 1).

### Stability studies in PBS, and mouse and human serum

The ^177^Lu-labeled peptides were incubated in 300-μL phosphate buffered saline (PBS; Gibco, ThermoFisher, The Netherlands) at 37 °C, and their stability was verified after 1, 4, 24, 48, and 72 h by radio-HPLC (Supplementary Fig. 2). The stability in serum was determined by incubating the radiolabeled compounds into 300 μL of mouse serum (Invitrogen, USA) or human serum (Sigma Aldrich, USA) at 37 °C (Supplementary Fig. 3, 4). At each time point post-incubation, 35 μL of the mixture was added to an Eppendorf tube, and the proteins were precipitated by adding an equal volume of acetonitrile. The tubes were centrifuged at 16,000 rpm, 4 °C for 20 min, and the stability was monitored by radio-HPLC, expressed as the RCP at the different time points**.**

### Cell culture

PC-3 cells were cultured in Ham’s F-K12 nutrient mix (Gibco, ThermoFisher, The Netherlands) supplemented with 10% fetal bovine serum (Gibco, ThermoFisher, The Netherlands), penicillin (100 IU/ml, Gibco, ThermoFisher, The Netherlands) and streptomycin (100 µg/ml, Gibco, ThermoFisher, The Netherlands) at 37 °C in a humidified atmosphere of 5% CO_2_ of air (NuAire).

### Competition binding assay and uptake

PC-3 cells were seeded at 2.5 × 10^5^ cells/well into 12 well plates 18–24 h prior to the experiment. Cells were incubated with 10^−9^ M of [^177^Lu]Lu-NeoB or [^177^Lu]Lu-RM2 together with increasing concentrations (10^−12^–10^−6^ M) of unlabeled NeoB, RM2, or Tyr^4^-bombesin (BBN; Sigma Aldrich, The Netherlands) (an analogue of natural occurring bombesin, thus an agonist) for 1 h at 37 °C. Hereafter, cells were washed twice with PBS and subsequently lysed with 0.1 M NaOH for 20 min at room temperature (RT). The cell lysates were collected and measured using a gamma counter (1480 WIZARD automatic γ-counter, PerkinElmer, The Netherlands). For the uptake studies, PC-3 cells (2.5 × 10^5^) were incubated with 10^−9^ M of [^177^Lu]Lu-NeoB or [^177^Lu]Lu-RM2 with or without 10^−6^ M Tyr^4^-BBN for 1 h at 37 °C. Hereafter, cells were washed twice with cold PBS and lysed with 0.1 M NaOH for 20 min at RT. The cell lysates were collected and measured to determine the amount of radioactivity in a gamma counter. To correct for cell density, cells from two separate wells were counted (Countess counter, Invitrogen, The Netherlands), and data was expressed as percentage of added dose (AD) per 2.5 × 10^5^ cells.

### Animal model

All animal experiments were approved by the Animal Welfare Committee of the Erasmus MC, and all experiments were conducted in accordance to institutional guidelines. Male NMRI-Foxn1 nu/nu mice (5 weeks old) were subcutaneously inoculated with 5 × 10^6^ PC-3 cells on the right shoulder (100 µL: 1/3 Matrigel (Corning, The Netherlands) and 2/3 Hank’s Balanced Salt Solution (Gibco, ThermoFisher, The Netherlands). Tumors were grown for 3 weeks resulting in an average volume of 362 ± 102 mm^3^.

### In vivo biodistribution

PC-3 tumor-bearing mice were intravenously injected with 200 µL of 9 MBq/150 pmol [^177^Lu]Lu-NeoB or [^177^Lu]Lu-RM2. At 1, 4, 24, 48, and 72 h post-injection (p.i.), animals were sedated using 2.5% isoflurane in oxygen, and blood was collected via the orbital vein, after which the animals were euthanized using cervical dislocation. Subsequently, the following organs were collected; the tail, tumor, spleen, liver, pancreas, small intestine, colon, coecum, kidneys, lung, and muscle. To assess in vivo specificity of [^177^Lu]Lu-NeoB and [^177^Lu]Lu-RM2, an additional group of tumor-bearing animals was injected with the radiolabeled peptides plus an 196-fold excess of Tyr^4^-BBN and biodistribution studies were performed as described above at 4 h p.i. The collected tumor and organs were weighed, and the radioactivity was measured in the gamma counter. The biodistribution data are expressed as percentage injected dose per gram of tissue (% ID/g). Animal numbers per group are indicated in Supplementary Table 1 and 2.

### SPECT/CT imaging

Whole-body single-photon emission computed tomography and computed tomography (SPECT/CT; VECTor/CT, MILabs, The Netherlands) were acquired in PC-3 tumor-bearing mice 1, 4, and 24 h p.i. of 9 MBq/150 pmol of [^177^Lu]Lu-NeoB or [^177^Lu]Lu-RM2 under sedation (2.5% isoflurane in oxygen) with body temperature maintained by a heating pad (*N* = 1 per radiotracer and per time point). The CT was obtained using the following parameters: 50 kV, 210 μA, 2 bed positions, and an acquisition time of 5 min. SPECT scans were acquired with the XXUHS-M, 3 mm collimator in list mode using 35 bed positions with an acquisition time of 30 min. Images were reconstructed using MILabs rec 12 using the SROSEM method with the following parameters: voxel size 0.4 mm; 9 iterations and 128 subsets; and 1 mm FWHM Gaussian filter. Post-analysis of the scans were performed using the Imalytics preclinical software (Gremse-IT, Germany). After each scan, animals were euthanized, and the organs were collected for biodistribution purposes, as described above.

### Autoradiography on human tumor and healthy tissue

Fresh frozen tissues of 3 human prostate cancers (PCa), breast cancers (BC), and gastrointestinal stromal tumors (GIST) were sectioned into 10-µm thick slices and mounted onto glass slides (ThermoFisher, The Netherlands). The tissue slices were pre-incubated with washing buffer (167 mM Tris–HCL (pH 7.6), 5 mM MgCl_2_) containing 0.25% BSA for 10 min at RT. Hereafter, tissue slides were incubated with 10^–9^ M of [^111^In]In-NeoB or [^111^In]In-RM2 with or without 10^−6^ M Tyr^4^-BBN for 1 h at RT. Following incubation, slides were washed 3 times, dried, and loaded into the BeaQuant (Atlantic Instruments for Research, France) for 48 h, and analyzed using Beamage software (Atlantic Instruments for Research, France). Quantified binding to tumor tissue was corrected for unspecific binding (quantified signal from blocked tissues). To allow comparison of separate experiments, the background signal was also quantified and subtracted. The data are expressed as % AD/mm^2^/min.

### Statistics

Statistical analysis was performed using Graphpad Prism version 9.0.0. Outliers were identified using the ROUT method with a *Q* value of 1%. IC_50_ curves were determined using non-linear regression. Significant differences between the IC_50_ and uptake values of radiolabeled NeoB and RM2 were determined using an unpaired *t* test. The tumor half-life of [^177^Lu]Lu-NeoB was determined using a non-linear regression followed by an uptake and excretion curve. To determine the tumor half-life of [^177^Lu]Lu-RM2 and the pancreas half-lives of both radiotracers a non-linear regression was applied followed by a one phase decay model. Significant differences between the tumor/organ uptake and tumor/organ half-lives of radiolabeled NeoB and RM2 derived from the biodistribution studies were determined using a 2-way ANOVA.

## Results

### Stability

[^177^Lu]Lu-NeoB and [^177^Lu]Lu-RM2 were obtained in > 98% RCY and > 97% RCP. Both radiotracers showed excellent stability in PBS up to 24 h at 37 °C (Table 1, Supplementary Fig. 2). However, after 48 h, [^177^Lu]Lu-RM2 stability in PBS slightly decreased in a time dependent manner while that of [^177^Lu]Lu-NeoB was maintained. Stability studies in mouse serum showed a decrease in stability over time for both [^177^Lu]Lu‐NeoB (94.3 ± 0.7% intact radiotracer at 1 h vs. 4.4 ± 1.2% at 72 h) and [^177^Lu]Lu‐RM2 (90.9 ± 0.5% intact radiotracer at 1 h vs. 5.3 ± 0.9% at 72 h) (Table 1, Supplementary Fig. 3). The stability of both [^177^Lu]Lu‐NeoB and [^177^Lu]Lu‐RM2 in human serum was noticeably superior to their stability in mouse serum (Table 1, Supplementary Fig. 4). [^177^Lu]Lu‐NeoB stability in human serum, however, was slightly higher at the 1 and 4 h time point, and slightly lower at the 24, 48, and 72 h time point.

**Table 1:** *S*tability in PBS, mouse and human serum of [^177^Lu]Lu-NeoB and [^177^Lu]Lu-RM2, (*N* = 3)*

		[^177^Lu]Lu-NeoB						[^177^Lu]Lu-RM2				
	0 h	1 h	4 h	24 h	48 h	72 h	0 h	1 h	4 h	24 h	48 h	72 h
PBS (%)	97.1 ± 0.3	97.8 ± 0.3	97.4 ± 0.5	96.3 ± 0.3	95.1 ± 0.4	93.8 ± 0.9	97.4 ± 0.3	97.8 ± 0.4	96.7 ± 0.5	95.6 ± 0.3	87.6 ± 0.6	80.4 ± 0.8
Mouse serum (%)		94.3 ± 0.7	84.1 ± 1.1	27.9 ± 2.3	14.2 ± 1.0	4.4 ± 1.2		90.9 ± 0.5	73.6. ± 0.4	29.6 ± 1.2	17.1 ± 3.5	5.3 ± 0.9
Human serum (%)		97.9 ± 0.2	97.8 ± 0.3	89.2 ± 0.5	81.0 ± 0.7	75.6 ± 1.7		96.7 ± 0.7	95.5 ± 1.1	94.9 ± 1.5	92.3 ± 1.9	88.4 ± 0.6

*Results are expressed as mean percentage ± SD of intact radiolabeled ligand (RCP) after incubation at 37 °C of three experiments

### In vitro competition binding and uptake

**Fig. 2:**
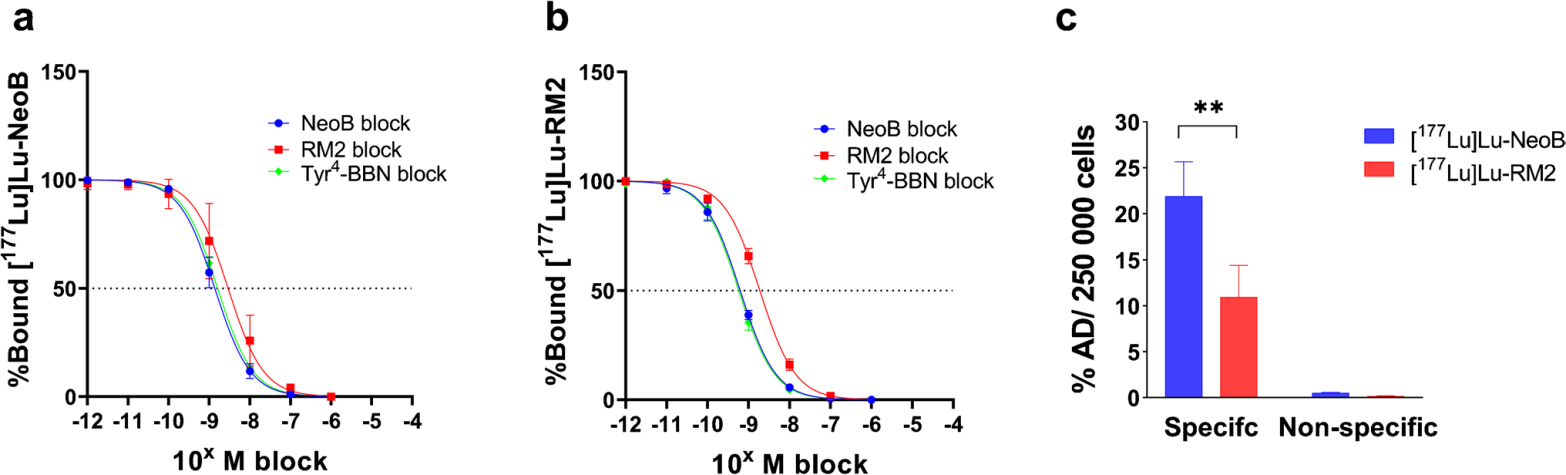
Binding of **a** [^177^Lu]Lu-NeoB and **b** [^177^Lu]Lu-RM2 in the presence of increasing concentrations of unlabeled NeoB, RM2, or Tyr^4^-BBN. **c** Specific (total) uptake shown for [^177^Lu]Lu-NeoB and [^177^Lu]Lu-RM2. Non-specific uptake was determined in the presence of a 1000 times excess of Tyr.^4^-BBN. Data are shown as mean %AD/250 000 cells ± SD. *P ≤ 0.05; **P ≤ 0.01; ***P ≤ 0.001

The competition binding assay was performed to determine the affinity of ^177^Lu-labeled NeoB and RM2. The concentration of unlabeled NeoB, RM2, and Tyr^4^-BBN needed to block 50% of ^177^Lu-labeled NeoB or RM2, were all in the nanomolar (nM) range (Fig. 2). However, more unlabeled NeoB, RM2 and Tyr^4^-BBN were required to block 50% of [^177^Lu]Lu-NeoB compared to the amount needed to block 50% of [^177^Lu]Lu-RM2 uptake (Table 2).

**Table 2:** IC_50_ values for unlabeled NeoB, RM2, and Tyr^4^-BBN as mean ± SD

	[^177^Lu]Lu-NeoB	[^177^Lu]Lu-RM2	*P* value
NeoB block	1.42 ± 0.41 nM	0.63 ± 0.04 nM	0.01
RM2 block	3.53 ± 2.36 nM	1.90 ± 0.31 nM	0.3
Tyr^4^-BBN block	1.61 ± 0.18 nM	0.58 ± 0.05 nM	< 0.001

*N* = At least 3

This was also reflected in the uptake of the radiotracers; the uptake of [^177^Lu]Lu-NeoB was 21.90 ± 3.76% AD, while this was 10.96 ± 3.46% AD for [^177^Lu]Lu-RM2 (Fig. 2c). For both [^177^Lu]Lu-NeoB and [^177^Lu]Lu-RM2, the uptake in the presence of Tyr^4^-BBN was low (< 1% AD), demonstrating specificity of binding.

### Biodistribution

**Fig. 3:**
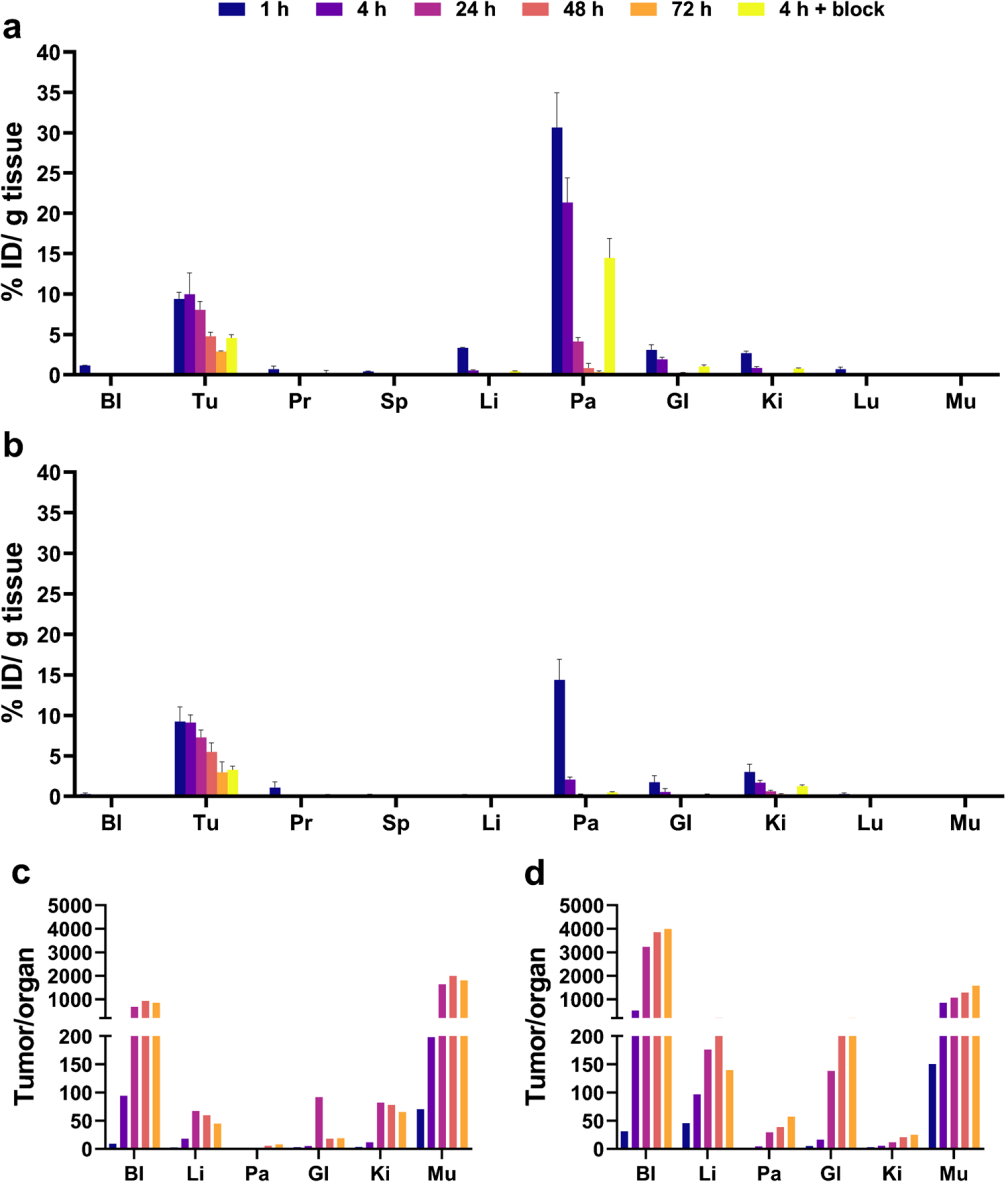
Biodistribution profile of **a** [^177^Lu]Lu-NeoB and **b** [^177^Lu]Lu-RM2 at 1, 4, 24, 48, 72 h p.i. of PC-3 tumor-bearing mice. Tumor to background ratio of organs of interest calculated from the biodistribution data of **c** [^177^Lu]Lu-NeoB and **d** [^177^Lu]Lu-RM2. Bl = blood; Tu = PC-3 tumor; Pr = prostate; Sp = spleen; Li = liver; Pa = pancreas; GI = gastrointestinal tract; Ki = kidney; Lu = lungs; Mu = muscle

Biodistribution studies were performed at 1, 4, 24, 48, and 72 h p.i. of [^177^Lu]Lu-NeoB or [^177^Lu]Lu-RM2 (Fig. 3A and B, Supplementary table 1 and 2). No significant differences were observed in tumor uptake of [^177^Lu]Lu-NeoB and [^177^Lu]Lu-RM2 at any time point. However, a difference in uptake in background organs was observed between the radiotracers, resulting in a difference in tumor-to-organ ratios. At all investigated time points, animals injected with [^177^Lu]Lu-NeoB had a lower tumor-to-blood, tumor-to-liver, tumor-to-pancreas, and tumor-to-gastrointestinal tract ratio, but a higher tumor-to-kidney ratio compared to animals that received [^177^Lu]Lu-RM2 (Fig. 3c and d). Among all collected organs, the GRPR-expressing pancreas showed the highest radiotracer uptake with both [^177^Lu]Lu-NeoB and [^177^Lu]Lu-RM2 at the 1 h time point, resulting in the lowest tumor-to-organ ratio for this organ. However, pancreatic uptake was significantly higher with [^177^Lu]Lu-NeoB compared to [^177^Lu]Lu-RM2. At the 1 h time point, the tumor and pancreatic uptake of [^177^Lu]Lu-NeoB was 9.38 ± 0.81% ID/g and 30.66 ± 4.29% ID/g, respectively, while for [^177^Lu]Lu-RM2 this was 9.27 ± 1.81% ID/g and 14.39 ± 2.56% ID/g, respectively (Supplementary table 1 and 2). The pancreas remained the organ with the highest uptake over time in animals that received [^177^Lu]Lu-NeoB, but this was not the case for animals that received [^177^Lu]Lu-RM2; the kidneys were the organ with the highest uptake from 24 p.i. onwards with [^177^Lu]Lu-RM2. Therefore, the tumor-to-pancreas ratios remained the most unfavorable for [^177^Lu]Lu-NeoB, but for [^177^Lu]Lu-RM2 the tumor-to-kidney ratio was lowest of all tumor-to-organ ratios at the 24, 48, and 72 h time points (Fig. 3c and d).

Blocking studies 4 h p.i. reduced tumor and pancreatic uptake of both radiotracers, but did not block uptake completely. For [^177^Lu]Lu-NeoB tumor uptake was 9.98 ± 2.64% ID/g vs. 4.55 ± 0.42% ID/g when Tyr^4^-BBN was co-injected, and pancreatic uptake was 21.35 ± 3.05% ID/g vs. 14.48 ± 2.40% ID/g, respectively. For [^177^Lu]Lu-RM2, this was 9.14 ± 0.94% ID/g vs. 3.27 ± 0.46% ID/g for the tumor, and 2.07 ± 0.30% ID/g vs. 0.49 ± 0.08% ID/g for the pancreas.

**Fig. 4:**
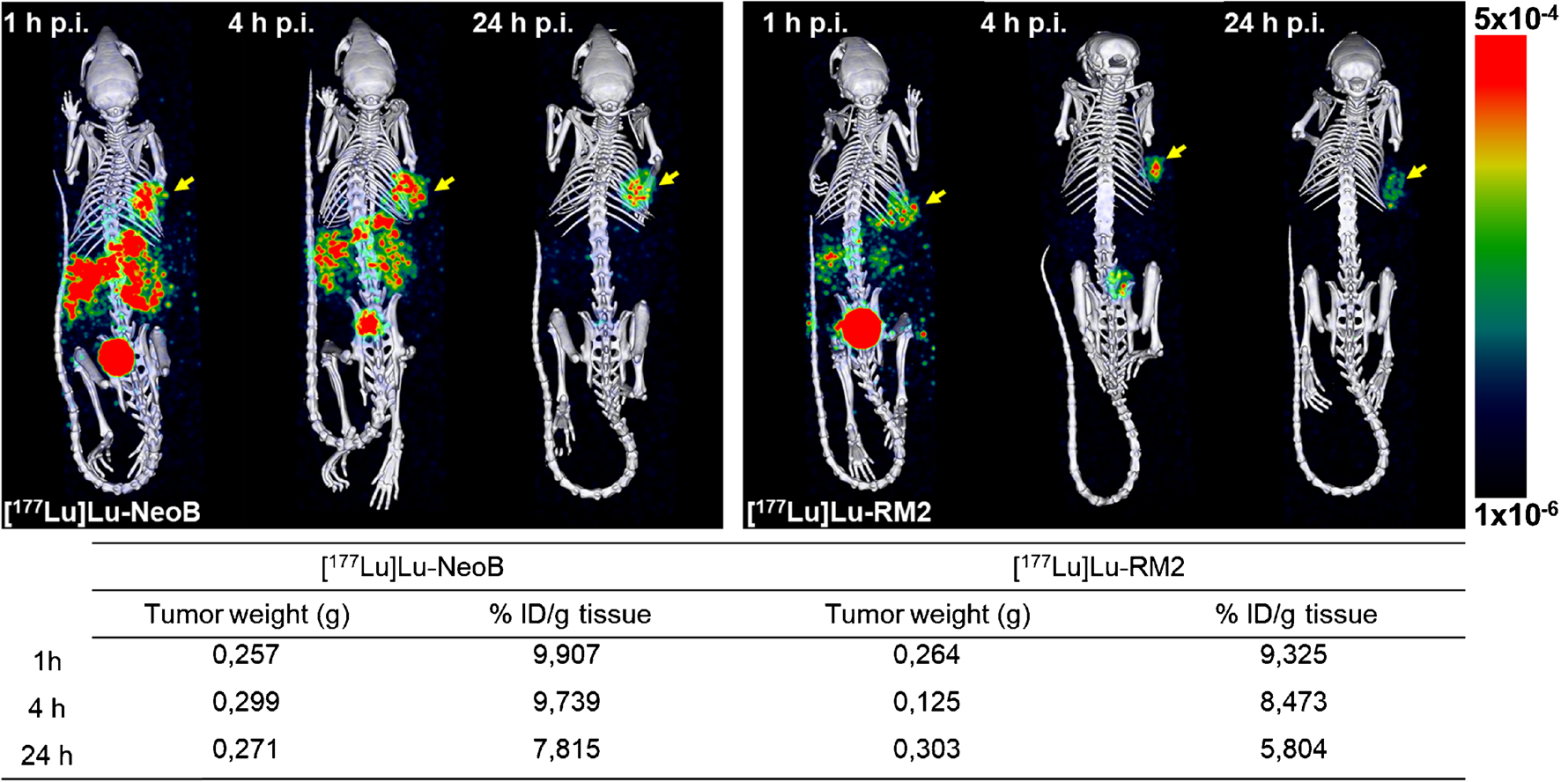
SPECT/CT images of PC-3 tumor-bearing mice 1, 4, and 24 h p.i. of (left) [^177^Lu]Lu-NeoB and (right) [^177^Lu]Lu-RM2. The tumor is located on the right shoulder and is indicated with a yellow arrow. *N* = 1

SPECT/CT images acquired at 1, 4, and 24 h p.i. of [^177^Lu]Lu-NeoB or [^177^Lu]Lu-RM2 were in line with the biodistribution data (Fig. 4). Due to the high uptake of both [^177^Lu]Lu-NeoB or [^177^Lu]Lu-RM2, the tumor could be visualized already at 1 h p.i. The only physiological organs visible at this time point were the pancreas, kidneys, and the bladder.

### Clearance

**Fig. 5:**
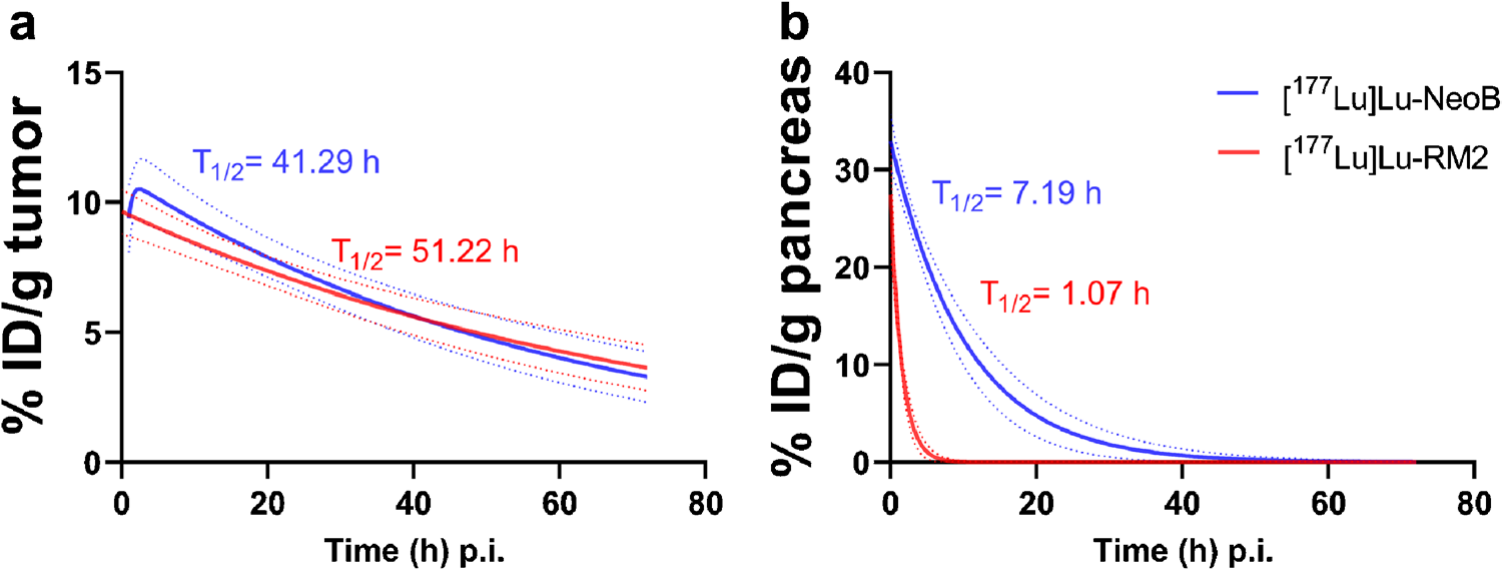
Pharmacokinetic modeling of the **a** tumor and **b** pancreas half-life clearance for [^177^Lu]Lu-NeoB and [^177^Lu]Lu-RM2

Despite the high pancreatic uptake of the radiotracers, clearance was fast. However, the pancreatic half-life of [^177^Lu]Lu-NeoB was significantly higher compared to that of RM2; 7.19 ± 2.09 h vs. 1.07 ± 0.29 h (*P* < 0.001), respectively. The tumor half-live was 41.29 ± 12.98 h for [^177^Lu]Lu-NeoB and 51.22 ± 15.17 h for [^177^Lu]Lu-RM2 (Fig. 5).

### Autoradiography

**Fig. 6:**
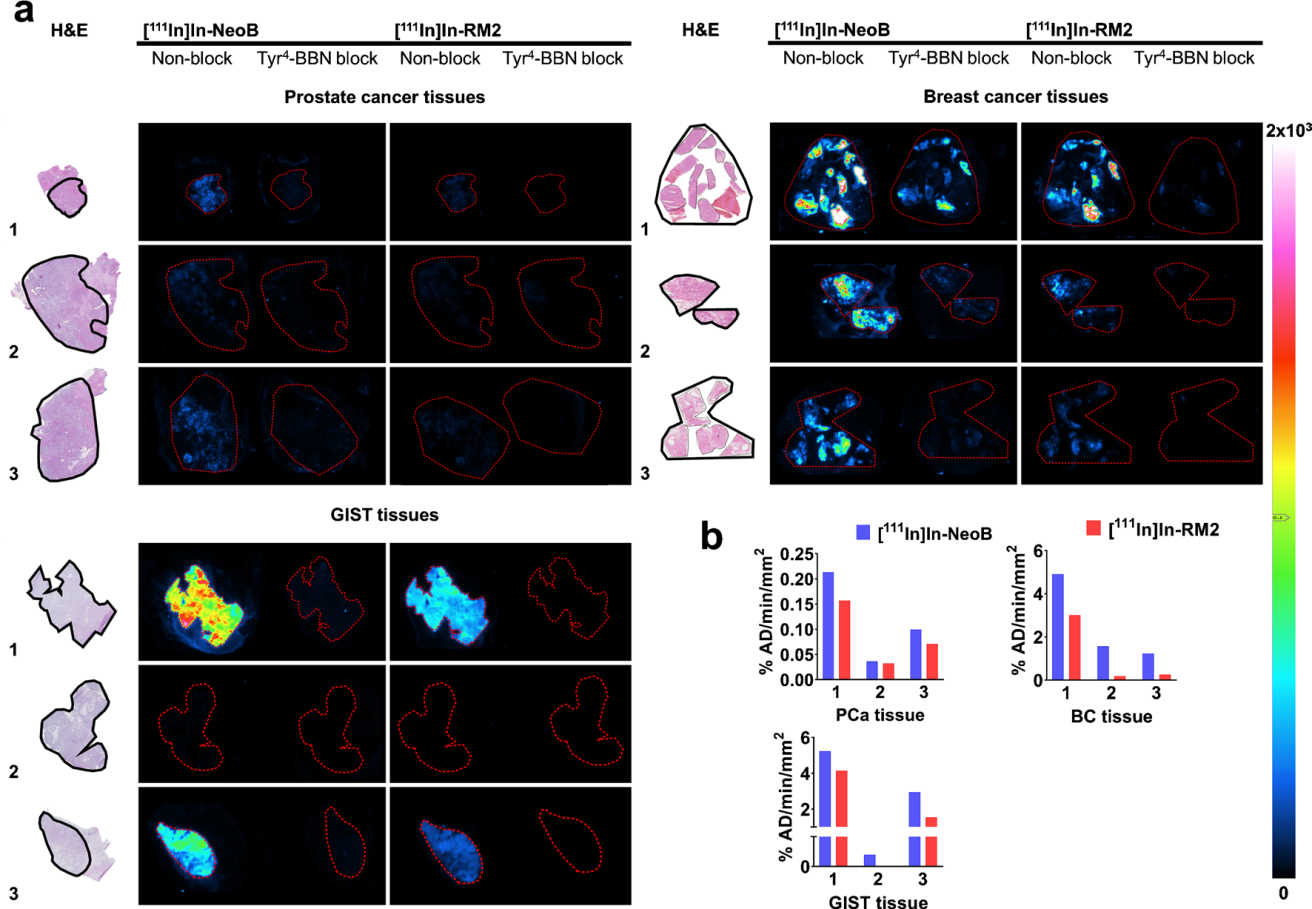
**a** In vitro autoradiography of [^111^In]In-NeoB and [^111^In]In-RM2 performed on GRPR-expressing human PCa, BC, GIST tumor tissues. Samples are from different patients. **b** % AD/min/mm^2^ calculated for the region of interests for marked on the H&E (black line) and the ARG (red dotted line) samples. Artefact hotspots were removed from the [^111^In]In-NeoB blocked PCa tissue 3

The autoradiography studies revealed a higher binding of [^111^In]In-NeoB to PCa, BC, and GIST tissues (Fig. 6). Tyr^4^-BBN did not completely block [^111^In]In-NeoB and [^111^In]In-RM2 in the BC tissues, while this was the case for PCa and GIST tissues.

## Discussion and conclusion

Several GRPR analogs have been and are being developed for targeted radionuclide imaging and PRRT of GRPR-expressing tumors. In order to guide future studies, we compared the two most studied GRPR-targeting radio-antagonists, NeoB and RM2. Overall, the stability of both radiotracers in PBS and human serum was high. The stability in mouse serum, however, decreased relatively fast over time. As both [^177^Lu]Lu-NeoB and [^177^Lu]Lu-RM2 are peptide-based molecules, which are known for their fast pharmacokinetics, it seems unlikely that the low percentage of intact radiotracer after 24 h impacts our results. In a recent study, Gunther et al. [25] also compared the stability of the two radiotracers. The authors reported [^177^Lu]Lu-NeoB to be more stable than [^177^Lu]Lu-RM2 in both human and mouse plasma. The differences observed in stability reported by Gunther et al. and the current study are most likely due to differences in the experimental setup. First, we used quenchers which have previously shown to prevent radiolysis [23]. Secondly, in the study by Gunther et al., radiotracer stability was determined in plasma, while we performed our stability studies in serum. Finally, in the previously published study, the stability in mouse plasma was determined in vivo (30 min p.i.), while in the current study the radiolabeled tracers were incubated in commercially available human and mouse serum in vitro.

We also demonstrated that both radiolabeled GRPR analogs bind with good affinity to their target. However, NeoB showed a slightly better GRPR affinity and accordingly radiolabeled NeoB had a higher uptake than radiolabeled RM2 in vitro, which is in accordance with the study by Gunther et al. [25]. Furthermore, our autoradiography study revealed higher binding of radiolabeled NeoB in all investigated human cancer samples. For this study, we have selected 3 cancer types which are known to have high GRPR expression [26]. In our PCa samples, however, we observed low binding of both radiotracers. Although PCa is commonly associated with high(er) GRPR expression, this may vary significantly depending on, among other factors, the disease stage. Nevertheless, we were able to demonstrate differences in binding of radiolabeled NeoB and RM2 on these cancer tissues.

The in vitro cell and tissue models provide information about the affinity and binding capabilities of the radiotracers, but do not take their pharmacokinetic properties into account. We have therefore included extensive biodistribution and imaging studies. The results showed that the more favorable GRPR affinity and higher uptake/binding of radiolabeled NeoB observed in vitro did not translate to higher tumor uptake of the radiotracer in vivo; [^177^Lu]Lu-NeoB and [^177^Lu]Lu-RM2 had similar tumor uptake values in vivo at all investigated time points. The difference in in vitro and in vivo uptake is most likely caused by the difference of the radiotracer pharmacokinetics in the two systems. Furthermore, the significantly higher pancreatic uptake of [^177^Lu]Lu-NeoB compared to [^177^Lu]Lu-RM2 might have led to lower [^177^Lu]Lu-NeoB tumor uptake than expected based on the in vitro findings.

During the in vitro cell studies, Tyr^4^-BBN successfully displaced [^177^Lu]Lu-NeoB and [^177^Lu]Lu-RM2 from GRPR binding sites on PC-3 cells, indicating specificity, but the molecule was less effective in blocking GRPR-mediated uptake of both radiotracers in vivo. Other studies reported similar findings when blocking with Tyr^4^-BBN in vivo [13, 14]. Tyr^4^-BBN is an agonist which triggers internalization of the receptor-ligand complex upon binding and potentially also recycling of the receptor back to the membrane hereafter. If the GRPR becomes available at the membrane again, the antagonistic radiotracers can again bind to the receptor [27, 28]. This can occur both in vitro and in vivo, but during our in vitro studies cells were incubated for only 1 h with the radiotracers while the in vivo block studies were performed 4 h p.i. of the radiotracers. The latter might be enough time for the proposed mechanism to occur. Future studies should use antagonists for blocking purposes. Regarding Tyr^4^-BBN, to confirm our hypothesis, studies should be performed at early and extended incubation periods.

In the autoradiography studies, Tyr^4^-BBN was also unable to completely block binding of the radiotracers to BC tissues. The high density of connective tissue present in these samples might have led to unspecific binding of the peptides to the tissues. NeoB, based on its structure and partition coefficient (LogD), is more lipophilic than RM2 [25, 29], which might have led to a higher unspecific binding of this radiotracer. In our autoradiography studies we corrected the quantified values for un-specific binding and thus the aforementioned does not influence the analysis with respect to comparison of binding of the two radiotracers to the evaluated human cancers.

In the biodistribution study, besides tumor and pancreatic uptake, low uptake in the liver, kidney, and gastrointestinal tract was observed with both [^177^Lu]Lu-NeoB and [^177^Lu]Lu-RM2. The tumor-to-liver ratio observed with [^177^Lu]Lu-NeoB was lower compared to that of [^177^Lu]Lu-RM2. Liver uptake of radiolabeled NeoB has also been reported by others and suggests hepatic clearance of the compound [30]. The radiolabeled NeoB molecules that are cleared hepatically might be those that were initially taken up by the pancreas. After being metabolized in the pancreas, they are carried to the liver via the hepatic portal vein for excretion [31, 32]. The lower pancreatic uptake and the faster pancreatic clearance of [^177^Lu]Lu-RM2 might explain why lower liver uptake was observed for [^177^Lu]Lu-RM2. Additionally, the observed liver uptake might also be linked to the lipophilicity of the compounds, as lipophilic compounds tend to have higher liver uptake.

Regarding the pancreatic uptake, the off target uptake in this organ is important when it comes to potential radiotoxicity during/after PRRT. The difference that was observed in pancreatic uptake between [^177^Lu]Lu-NeoB and [^177^Lu]Lu-RM2, resulting in a more favorable tumor-to-pancreas ratio for [^177^Lu]Lu-RM2, can potentially be explained by a difference in affinity for the murine GRPR between the two radiotracers. In an attempt to elucidate the above, we performed autoradiography studies on mouse and human pancreas tissue. However, only slight binding was observed to the mouse pancreas tissue, and no binding was observed to human pancreas tissue (data for mouse pancreas shown in Supplementary Fig. 5). This is most likely because the receptor was only present at low density or not present at all on the cells of the mouse and human pancreas, respectively; at the moment, the analysis was performed. As the organ is full of enzymes, the GRPR is most likely degraded enzymatically during tissue collection and storage. In addition to the difference in affinity for the mouse and human GRPR, factors such as microvasculature and perfusion [33] can also cause a discrepancy in results obtained in mice vs. men.

Moreover, whether or not the pancreas is at risk and should be considered as a dose-limiting organ in GRPR-mediated PRRT has been investigated by several groups. Even though we did not study the toxicity of the radiotracers in the current study, the pancreatic half-life of the radiotracers observed suggests that the dose to which the organ is exposed is limited. Furthermore, in a recently published clinical study by Kurth et al., it was reported that [^177^Lu]Lu-RM2 was well tolerated by all patients [20]. The authors reported that [^177^Lu]Lu-RM2 showed high uptake in the tumor and cleared rapidly from normal organs, including the pancreas. Regarding NeoB, a pre-clinical study has shown [^177^Lu]Lu-NeoB to be well tolerated after repeated radiotracer administration. In the above-mentioned study, radiotoxic effects were found in the kidneys [30]. In our study, a more unfavorable tumor-to-kidney ratio was observed with [^177^Lu]Lu-RM2 compared to [^177^Lu]Lu-NeoB; however, the kidney uptake was only significantly higher for [^177^Lu]Lu-RM2 at the 4 h time point. Extensive dosimetry studies should be performed to demonstrate whether or not the dose to which the kidneys is exposed is relevantly higher with [^177^Lu]Lu-RM2 vs. [^177^Lu]Lu-NeoB.

In addition, a lower tumor-to-blood ratio was observed for [^177^Lu]Lu-NeoB vs. [^177^Lu]Lu-RM2, indicating a faster clearance of [^177^Lu]Lu-RM2 from the blood. As next to the kidneys, bone marrow is often a dose-limiting organ, extensive dosimetry studies should also be performed to determine whether this difference observed between the radiotracers is relevant. Of note, only at the earliest time points (1 h and 4 h), when radiotracer uptake was relatively high in the blood, differences in blood uptake was significant between the radiotracers. It should also be mentioned that in the previously mentioned preclinical study evaluating [^177^Lu]Lu-NeoB toxicity, no lasting hematological effects were observed after treatment [30].The tumor-to-organ ratios, except the tumor-to-kidney ratios observed between [^177^Lu]Lu-NeoB and [^177^Lu]Lu-RM2 in this study, showed similar patterns as those calculated in the study of Gunther et al. [25]. However, in the study of Gunther et al., the tumor-to-kidney ratio for [^177^Lu]Lu-NeoB and [^177^Lu]Lu-RM2 were similar. This discrepancy might be a consequence of differences in experimental setup (e.g., labeling, injected dose, mouse strain) between our study and theirs. Strategies to decrease physiologic uptake in the background organs and increase tumor uptake are being evaluated by various groups. Wang et al., developed a series of GRPR antagonists (TacsBOMB1-5) based on the following sequence [Leu^13^ψThz^14^]Bombesin(7–14) of which TacsBOMB5 demonstrated significantly higher PC-3 tumor uptake and lower pancreatic uptake than RM2 [34]. It should be noted that this study was performed in a mouse models and might be different in humans, e.g., a difference in affinity for the murine and human GRPR might be at play. Another promising approach to reduce pancreatic uptake involves bioorthogonal chemistry, also known as the pretargeting strategy. Compared to conventional targeted therapies, pretargeting leads to higher tumor/background ratios, reduced circulation time of radioactivity, and facilitates the use of short-lived radionuclides [35–37]. D’Onofrio et al. developed a GRPR pretargeting radiocomplex which has successfully reduced pancreatic uptake [36]. However, uptake in the tumor was also diminished due to rapid washout and fast metabolization of the compound in vivo. Currently, pretargeting radiocomplexes with higher stability and longer circulation time are being developed.

To our knowledge, there is no head-to-head comparison of radiolabeled NeoB and RM2 in patients and comparing results of separately performed studies is challenging due to methodological differences and differences in the patient’s characteristics. Despite this, we attempted to compare dosimetry calculations reported in two clinical studies using [^68^ Ga]Ga-NeoB and [^68^ Ga]Ga-RM2 [38, 39]. In this comparison, the reported dose delivered to the pancreas, liver, and bladder was higher with radiolabeled NeoB vs. radiolabeled RM2. Furthermore, the analysis revealed no difference in kidney uptake between radiolabeled NeoB and RM2. The findings of this indirect comparison are in line with our study, with regards to a higher pancreatic and liver uptake with radiolabeled NeoB compared to radiolabeled RM2, and partly also for the kidney uptake as previously mentioned we only observed a significant difference in kidney uptake at the 4 h time point. However, a direct clinical comparison of the radiotracers is necessary to confirm these findings. Furthermore, the above findings might suggest that if a difference in affinity for the murine and human GRPR between the two radiotracers exists, this only partly explains our in vivo findings as also in humans’ radiolabeled NeoB results in a higher pancreatic dose.

To summarize, our studies demonstrated good stability of the two GRPR radiotracers up to 24 h, and specific and high uptake of the radiotracers in vitro and in vivo. Regarding the latter, uptake/binding was higher for radiolabeled NeoB in vitro on PC-3 cells and human cancer tissues, but no differences in tumor uptake was observed in vivo in PC-3 xenografts. Both radiotracers showed pancreatic uptake, but this was significantly lower for [^177^Lu]Lu-RM2. Additionally, the pancreas half-life was faster for this radiotracer. Moreover, a lower tumor-to-blood ratio was observed for [^177^Lu]Lu-NeoB, while tumor-to-kidney was lower for [^177^Lu]Lu-RM2. Based on these findings, we conclude that the in vivo tumor targeting capability of radiolabeled NeoB and RM2 are similar. However, patient studies should confirm whether the differences in uptake in background organs (e.g., pancreas, liver, kidney, blood) and pharmacokinetics are similar in humans, as this can be an important factor to consider when applying the radiotracers for PRRT.

### Supplementary Information

Below is the link to the electronic supplementary material.

Supplementary file1 (DOCX 646 kb)

## Data Availability

The datasets generated during the current study are available from the corresponding author on reasonable request.
