# Supplementary material for: Side-by-side comparison of the two widely studied GRPR radiotracers, radiolabeled NeoB and RM2, in a preclinical setting
Source: Eur J Nucl Med Mol Imaging. 2023 Aug 16;50(13):3851–61. doi: 10.1007/s00259-023-06364-4 (PMC10611828; doi:10.1007/s00259-023-06364-4)
Supplement: Supplementary file 1 — Supplementary file1 (DOCX 646 kb) [file 259_2023_6364_MOESM1_ESM.docx]

Supplementary information

**Side-by-side comparison of the two widely studied GRPR radiotracers, radiolabeled NeoB and RM2, in a preclinical setting**

T.S.T. Damiana, P. Paraïso, C. de Ridder, D. Stuurman, Y. Seimbille, S.U. Dalm

Department of Radiology & Nuclear Medicine, Erasmus Medical Center, Rotterdam, The Netherlands

Corresponding author: S.U. Dalm , s.dalm@erasmusmc.nl

# Material and method

## Supplementary material 1

#### Chemistry General Information

The chemicals and solvents were purchased from commercial suppliers and used without further purification. The Fmoc-based solid-phase peptide synthesis (SPPS) was performed manually in dedicated reaction vessels (Chemglass, Vineland, NJ, USA). Chlorotrityl resin and rink amide 4-methyl-benzhydrylalanine (MBHA) resin and all the Fmoc- protected amino acids were obtained from NovaBiochem (Laeufelfingen, Switzerland), Fmoc-4-amino-1-carboxymethylpiperidine from NeoMPS (Strasbourg, France), and DOTA-tris(*t*Bu)ester from Macrocyclics (Plano, TX, USA). Lutetium-177 (LuMark® Lutetium-177 chloride) was obtained from IDB Holland (Baarle-Nassau, The Netherlands). High-performance liquid chromatography (HPLC) and mass spectrometry (MS) were carried out on an LC/MS 1260 Infinity II system from Agilent (Middelburg, The Netherlands). Analyses were performed on an analytical column (Poroshell 120, EC-C18, 2.7 µm, 3.0 × 100 mm) from Agilent (eluents, A: water with 0.1 % formic acid (FA) and B: acetonitrile with 0.1 % FA; gradient: 0−8 min, 5−100 % B; flowrate: 0.5 mL/min). Purification of RM2 and NeoB was carried out on a preparative HPLC 1290 Infinity II system from Agilent (Middelburg, The Netherlands) using a preparative column (50 × 21.2 mm, 5 µm) from Agilent. Activity measurements were performed using a VDC-405 dose calibrator (Comecer; Joure, The Netherlands). Quality control of the radiolabeled compounds and analysis of their stability were carried out on an ultra-high performance liquid chromatography (UHPLC) Acquity Arc system from Waters (Etten-Leur, The Netherlands) equipped with a diode array detector and a radio-detector from Canberra (Zelik, Belgium). Analyses were performed on an analytical C18 Gemini® column (250.0 × 4.6 mm, 5 µm) from Phenomenex (CA, USA) eluted with a gradient (5−95 % B) of solvent A (water with 0.1 % trifluoroacetic acid (TFA)) and solvent B (acetonitrile with 0.1 % TFA) over 30 min at a flowrate of 1.0 mL/min.

#### Synthesis of DOTA-pADA-D-Phe-Gln-Trp-Ala-Val-Gly-His-NH-CH[CH_2_-CH(CH_3_)_2_]_2_ (**NeoB**)

NeoB was synthesized by standard Fmoc solid-phase peptide synthesis protocols. Chlorotrityl resin (0.25 g, average loading capacity: 1.6 mmol/g) was swelled in DCM. The peptide synthesis started by loading Fmoc-His(Trt)-OH (4 equiv.) preactivated with DIPEA (0.8 M in DMF, 8 equiv.) onto the resin. Subsequent Fmoc deprotection and coupling with Fmoc-Gly-OH (4 equiv.), Fmoc-Val-OH (4 equiv.), Fmoc-Ala-OH (4 equiv.), Fmoc-Trp(Boc)-OH (4 equiv.), Fmoc-Gln(Trt)-OH (4 eq) and Fmoc-D-Phe-OH (4 equiv.) were achieved. All coupling reactions were carried out in DMF with HBTU (3.9 equiv.), Oxyma Pure (4 equiv.) and DIPEA (8 equiv.) for 2 h. Fmoc deprotection are accomplished by treatment of the resin with a 20% solution of 4-methylpiperidine (4-MePip) in DMF (8 mL) for 15 min twice. Using a similar procedure, Fmoc-*p*-aminomethylaniline-diglycolic acid (Fmoc-*p*ADA-OH; 3 equiv.) was coupled onto the AA sequence overnight. Then, 1-(acetic acid)-4,7,10-tris(*tert*-butoxycarbonylmethyl)-1,4,7,10-tetraazacyclododecane (DOTA-tris(*t*Bu)ester, 3 equiv.) was conjugated to the peptide in presence of PyBOP (3 equiv.) and DIPEA (8 equiv.) in DMF (4 mL) overnight. Treatment of the resin with 1,1,1,3,3,3-hexafluoro-2-propanol (HFIP), triethylsilane (TES) and DCM in a ratio 20/5/75 v/v/v (8 mL total volume) for 15 min at room temperature (RT) gave the crude protected “NeoB”, which reacted with 4-amino-2,6-dimethylheptane in presence of PyBOP (3 equiv.) and DIPEA (8 equiv.) in DMF (5 mL). All protecting groups were removed in solution by treatment with a cocktail of trifluoroacetic acid (TFA), triisopropylsilane (TIPS) and water in a ratio 95:2.5:2.5 v/v/v (8 mL total volume)at RT for 3 h. Finally, the crude NeoB was purified by HPLC (eluents, A: water with 0.1 % FA and B: acetonitrile with 0.1 % FA; gradient: 0−12 min, 5−40 % B; flowrate: 10 mL/min). NeoB was obtained as a white solid (191 mg, 50.2%). *t*_R_ = 4.33 min; Purity > 95%. ESI-MS: m/z, calculated for C_77_H_110_N_18_O_18_: 1575.82, found: 1576.70 [M + H]^+^. **Supplementary Fig** 1a shows the chemical purity of NeoB.

#### Synthesis of DOTA-Pip-D-Phe-Gln-Trp-Ala-Val-Gly-His-Sta-Leu-NH_2_ (**RM2**)

Rink Amide MBHA Resin (0.13 g, average loading capacity: 0.642 mmol/g) was swelled in DCM and deprotected using a 20% 4-MePip solution in DMF (2 mL). The peptide synthesis started by loading Fmoc-Leu-OH (4 equiv.) preactivated with DIPEA (0.8 M in DMF, 8 equiv.) onto the resin. Subsequent Fmoc deprotection and coupling with Fmoc-Sta-OH (4 equiv.), Fmoc-His(Trt)-OH (4 equiv.), Fmoc-Gly-OH (4 equiv.), Fmoc-Val-OH (4 equiv.), Fmoc-Ala-OH (4 equiv.), Fmoc-Trp(Boc)-OH (4 equiv.), Fmoc-Gln(Trt)-OH (4 equiv.) and Fmoc-D-Phe-OH (4 equiv.) were achieved. All coupling reactions were carried out in DMF (1 mL) with HBTU (3.9 equiv.), Oxyma Pure (4 equiv) and DIPEA (8 equiv.) for 2 h. Fmoc deprotection are accomplished by treatment of the resin with a 20% solution of 4-MePip in DMF (2 mL) for 15 min twice. Using a similar procedure, Fmoc-4-amino-(1-carboxymethyl)piperidine (Fmoc-Pip-OH; 3 equiv.) was coupled onto the peptidyl resin, followed by the coupling of DOTA-tris(*t*Bu)ester (3 equiv.) in presence of PyBOP (3 equiv.) and DIPEA (8 equiv.) in DMF (2 mL) overnight to obtain the protected RM2. Finally, all protecting groups were removed by treatment with a cocktail of 2 mL TFA/TIPS/water (95:2.5:2.5 v/v/v) at RT for 3 h. Finally, the crude RM2 was purified by HPLC (eluents, A: water with 0.1 % FA and B: acetonitrile with 0.1 % FA; gradient: 0−12 min, 5−30 % B; flowrate: 10 mL/min). RM2 was obtained as a white solid (76.5 mg, 55.9 %). *t*_R_ = 3.59 min; Purity > 95%. ESI-MS: m/z, calculated for C_78_H_118_N_20_O_19_: 1639.92, found:1640.70 [M + H]^+^. **Supplementary Fig 1b** shows the chemical purity of RM2.

# Results

## Supplementary Figures


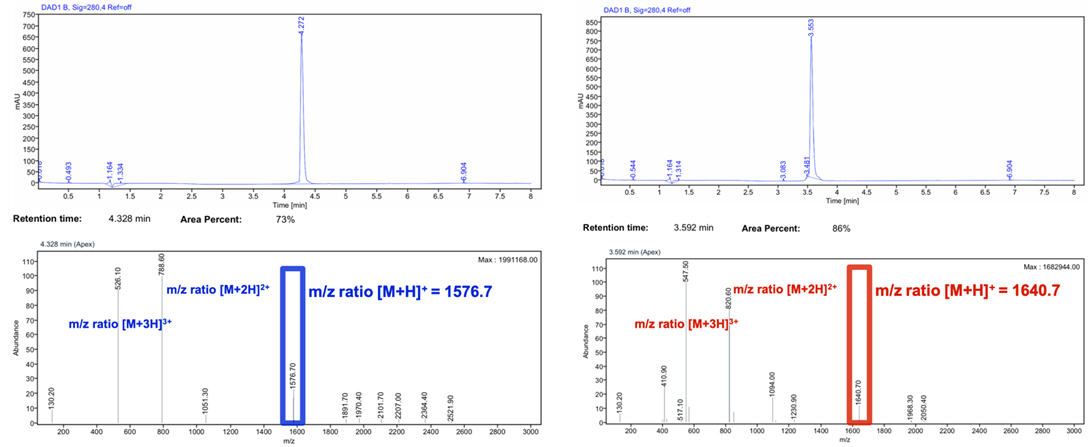


**Supplementary Fig 1**  HPLC chromatograms and mass spectra of (A) NeoB (left) and (B) RM2 (right)


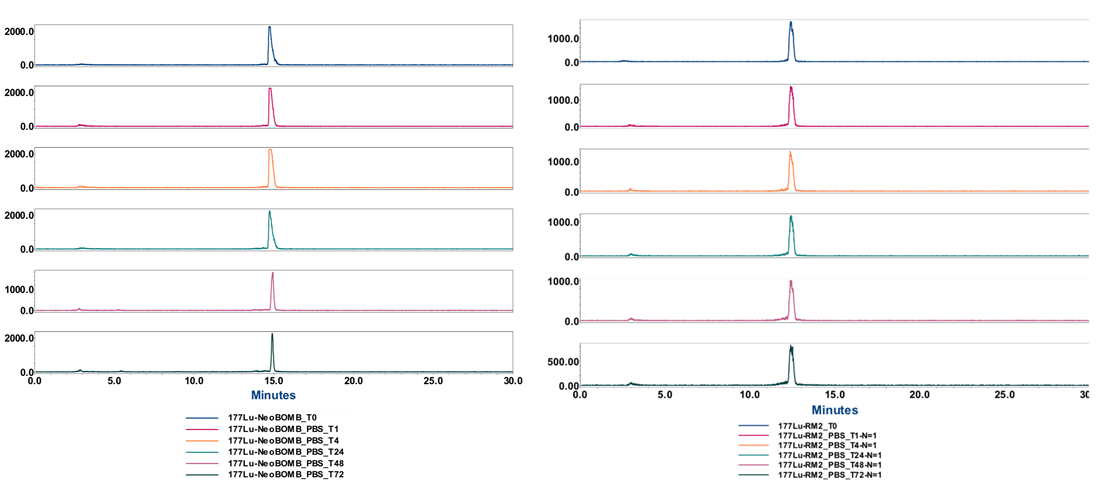


**Supplementary Fig 2** Radio-HPLC chromatograms of the stability studies performed for [^177^Lu]Lu-NeoB (left) and [^177^Lu]Lu-RM2 (right) at 1, 4, 24, 48 and 72 h post-incubation at 37˚C in PBS


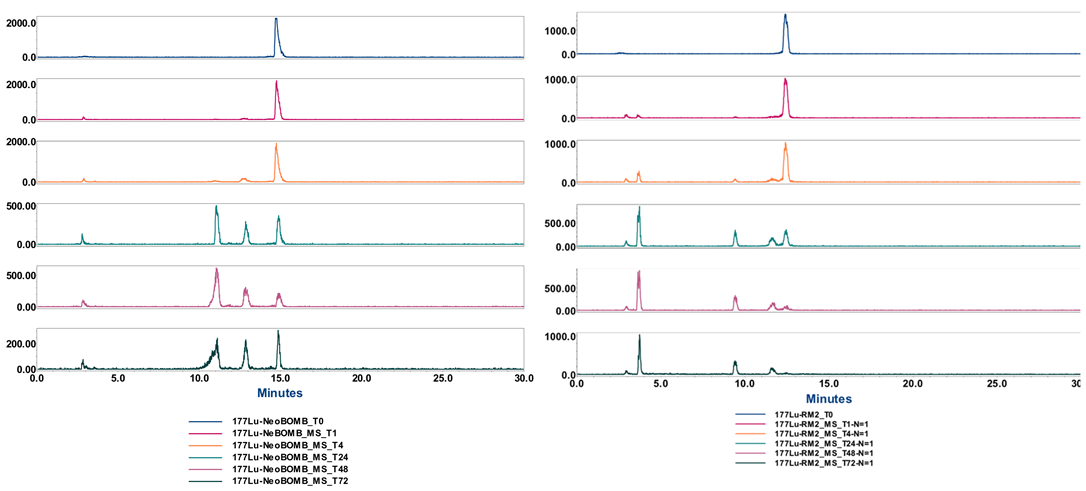


**Supplementary Fig 3.** Radio-HPLC chromatograms of the stability studies performed for [^177^Lu]Lu-NeoB (left) and [^177^Lu]Lu-RM2 (right) at 1, 4, 24, 48 and 72 h post-incubation at 37^˚^C in mouse serum.


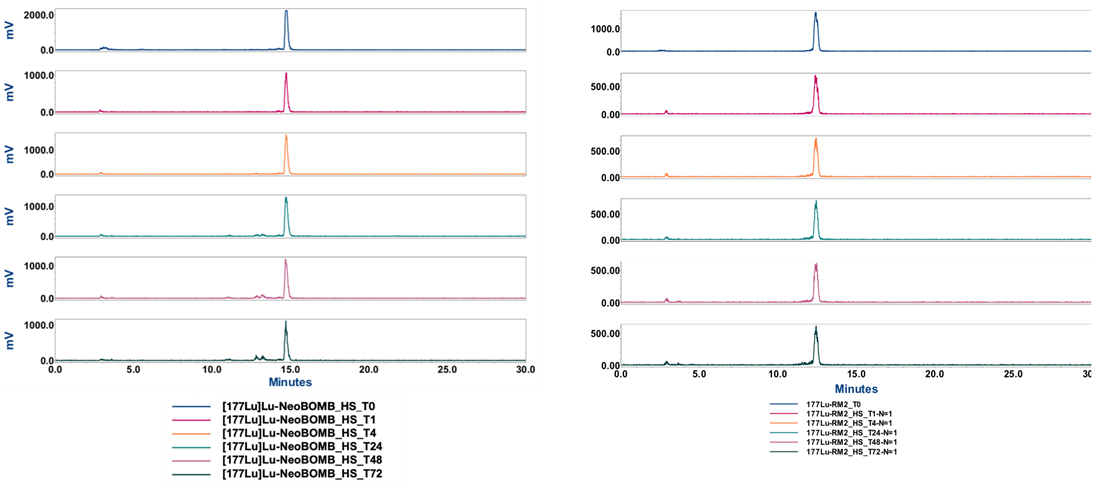


**Supplementary Fig 4** Radio-HPLC chromatograms of the stability studies performed for [^177^Lu]Lu-NeoB (left) and [^177^Lu]Lu-RM2 (right) at 1, 4, 24, 48 and 72 h post-incubation at 37^˚^C in human serum

**
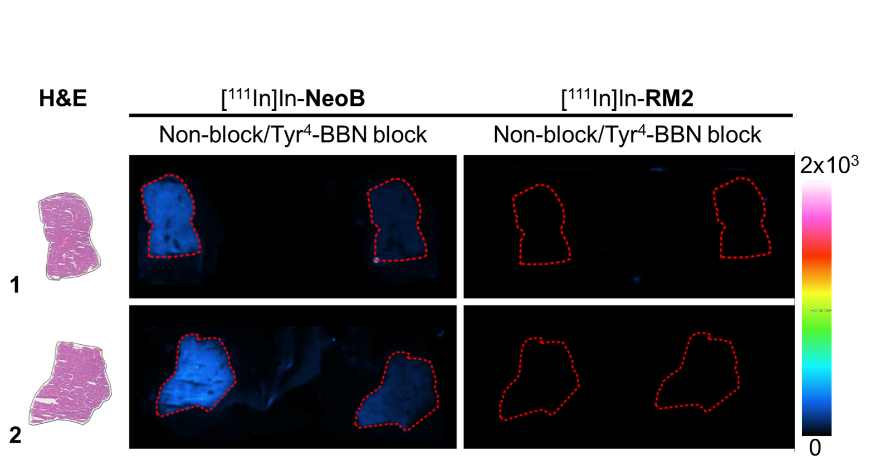
**

**Supplementary Fig 5** In vitro autoradiography of [^111^In]In-NeoB and [^111^In]In-RM2 performed PC-3 tumor and mice pancreas. Samples from two different mice were used. The region of interest for each sample is marked on the H&E (black line) and the ARG (red dotted line) samples

## Supplementary Tables

**Supplementary table 1**. Biodistribution data of [^177^Lu]Lu-NeoB at 1, 4, 24, 48, 72 h p.i. of PC-3 tumor-bearing mice.

|  | **1 h** | | | **4 h** | | | **4 h + block** | | | **24 h** | | | **48 h** | | | **72 h** | | |
| --- | --- | --- | --- | --- | --- | --- | --- | --- | --- | --- | --- | --- | --- | --- | --- | --- | --- | --- |
|  | **N=5 in total** | | | **N=5 in total** | | | **N=4 in total** | | | **N=5 in total** | | | **N=4 in total** | | | **N=4 in total** | | |
|  | % uptake | SD | N* | % uptake | SD | N* | % uptake | SD | N | % uptake | SD | N* | % uptake | SD | N | % uptake | SD | N |
| **Bl** | 1.15 | 0.03 | 4 | 0.11 | 0.02 | 5 | 0.08 | 0.01 | 4 | 0.01 | 0.00 | 5 | 0.01 | 0.00 | 4 | 0.00 | 0.00 | 4 |
| **Tu** | 9.38 | 0.81 | 5 | 9.98 | 2.64 | 5 | 4.55 | 0.42 | 4 | 8.05 | 1.04 | 5 | 4.77 | 0.49 | 4 | 2.88 | 0.08 | 4 |
| **Pr** | 0.70 | 0.39 | 5 | 0.08 | 0.02 | 4 | 0.23 | 0.30 | 4 | 0.02 | 0.01 | 5 | 0.01 | 0.01 | 4 | 0.01 | 0.01 | 4 |
| **Sp** | 0.43 | 0.05 | 5 | 0.15 | 0.02 | 5 | 0.14 | 0.02 | 4 | 0.04 | 0.00 | 5 | 0.02 | 0.01 | 4 | 0.01 | 0.00 | 4 |
| **Li** | 3.35 | 0.05 | 4 | 0.54 | 0.08 | 5 | 0.45 | 0.04 | 4 | 0.12 | 0.02 | 5 | 0.08 | 0.01 | 4 | 0.06 | 0.01 | 4 |
| **Pa** | 30.66 | 4.29 | 5 | 21.35 | 3.05 | 5 | 14.48 | 2.40 | 4 | 4.14 | 0.47 | 5 | 0.83 | 0.59 | 4 | 0.35 | 0.14 | 4 |
| **GI** | 3.08 | 0.63 | 5 | 1.91 | 0.27 | 5 | 1.03 | 0.20 | 4 | 0.09 | 0.04 | 4 | 0.26 | 0.04 | 4 | 0.15 | 0.02 | 4 |
| **Ki** | 2.68 | 0.25 | 5 | 0.86 | 0.17 | 5 | 0.78 | 0.07 | 4 | 0.10 | 0.03 | 5 | 0.06 | 0.00 | 4 | 0.04 | 0.01 | 4 |
| **Lu** | 0.69 | 0.26 | 5 | 0.09 | 0.03 | 5 | 0.07 | 0.01 | 4 | 0.02 | 0.00 | 5 | 0.01 | 0.00 | 3 | 0.01 | 0.00 | 4 |
| **Mu** | 0.13 | 0.03 | 5 | 0.05 | 0.05 | 5 | 0.02 | 0.00 | 4 | 0.00 | 0.00 | 5 | 0.00 | 0.00 | 4 | 0.00 | 0.00 | 4 |

**Bl** = blood; **Tu** = PC-3 tumor; **Pr**= prostate; **Sp** = spleen; **Li** = liver; **Pa** = pancreas; **GI** = gastrointestinal tract; **Ki** = kidney; **Lu** = lungs; **Mu** = muscle. *Number of organs included for analysis after performing an outlier test as indicated in the statistics paragraph of the Materials and Methods section

**Supplementary table 2.** Biodistribution data of [^177^Lu]Lu-RM2 at 1, 4, 24, 48, 72 h p.i. of PC-3 tumor-bearing mice.

|  | **1 h** | | | **4 h** | | | **4 h + block** | | | **24 h** | | | **48 h** | | | **72 h** | | |
| --- | --- | --- | --- | --- | --- | --- | --- | --- | --- | --- | --- | --- | --- | --- | --- | --- | --- | --- |
|  | **N=5 in total** | | | **N=5 in total** | | | **N=4 in total** | | | **N=5 in total** | | | **N=4 in total** | | | **N=4 in total** | | |
|  | % uptake | SD | N* | % uptake | SD | N | % uptake | SD | N | % uptake | SD | N | % uptake | SD | N | % uptake | SD | N |
| **Bl** | 0.30 | 0.13 | 5 | 0.02 | 0.01 | 4 | 0.01 | 0.01 | 4 | 0.00 | 0.00 | 5 | 0.00 | 0.00 | 4 | 0.00 | 0.00 | 4 |
| **Tu** | 9.27 | 1.81 | 5 | 9.14 | 0.94 | 5 | 3.27 | 0.46 | 4 | 7.32 | 0.91 | 5 | 5.47 | 1.13 | 4 | 2.97 | 1.29 | 4 |
| **Pr** | 1.08 | 0.71 | 5 | 0.03 | 0.02 | 4 | 0.11 | 0.12 | 3 | 0.03 | 0.01 | 5 | 0.02 | 0.00 | 4 | 0.01 | 0.00 | 4 |
| **Sp** | 0.19 | 0.08 | 5 | 0.06 | 0.01 | 5 | 0.09 | 0.04 | 4 | 0.04 | 0.01 | 5 | 0.03 | 0.01 | 4 | 0.02 | 0.01 | 4 |
| **Li** | 0.20 | 0.05 | 5 | 0.09 | 0.00 | 5 | 0.03 | 0.14 | 4 | 0.04 | 0.00 | 5 | 0.03 | 0.00 | 3 | 0.02 | 0.00 | 3 |
| **Pa** | 14.39 | 2.56 | 5 | 2.07 | 0.30 | 5 | 0.49 | 0.08 | 4 | 0.25 | 0.04 | 5 | 0.14 | 0.04 | 4 | 0.05 | 0.00 | 4 |
| **GI** | 1.75 | 0.79 | 5 | 0.56 | 0.40 | 5 | 0.18 | 0.10 | 4 | 0.05 | 0.01 | 4 | 0.03 | 0.01 | 4 | 0.01 | 0.00 | 4 |
| **Ki** | 3.00 | 0.95 | 5 | 1.69 | 0.27 | 5 | 1.25 | 0.17 | 4 | 0.63 | 0.13 | 5 | 0.26 | 0.09 | 4 | 0.12 | 0.02 | 4 |
| **Lu** | 0.30 | 0.12 | 5 | 0.04 | 0.01 | 5 | 0.05 | 0.02 | 4 | 0.02 | 0.00 | 5 | 0.01 | 0.00 | 4 | 0.01 | 0.00 | 4 |
| **Mu** | 0.06 | 0.03 | 5 | 0.01 | 0.00 | 4 | 0.03 | 0.01 | 4 | 0.01 | 0.00 | 5 | 0.00 | 0.00 | 4 | 0.00 | 0.00 | 4 |

**Bl** = blood; **Tu** = PC-3 tumor; **Pr**= prostate; **Sp** = spleen; **Li** = liver; **Pa** = pancreas; **GI** = gastrointestinal tract; **Ki** = kidney; **Lu** = lungs; **Mu** = muscle. *Number of organs included for analysis after performing an outlier test as indicated in the statistics paragraph of the Materials and Methods section
